# Neonatal portal vein thrombosis: risk factors, diagnosis, treatment recommendations and review of the literature

**DOI:** 10.1186/s12959-023-00508-0

**Published:** 2023-06-05

**Authors:** Huseyin Avni Solgun, Ezgi Paslı Uysalol, Cengiz Bayram, Özlem Terzi, Merih Çetinkaya, Şeyma Memur, Ali Aycicek

**Affiliations:** 1 Department of Pediatric Hematology and Oncology, Health Sciences University, Basaksehir Cam and Sakura Training and Research Hospital, Istanbul, Turkey; 2 Department of Neonatology, Health Sciences University, Basaksehir Cam and Sakura Training and Research Hospital, Istanbul, Turkey

**Keywords:** Neonatal, Portal vein thrombosis, Outcomes, Risk factors, Demographic features, Clinical features

## Abstract

**Background:**

Neonatal portal vein thrombosis (PVT) is currently more commonly encountered as a result of advances in diagnostic tools and increase in invasive interventions.

**Methods:**

In this study, 11 premature and 12 term infants diagnosed with PVT were retrospectively evaluated for clinical and laboratory characteristics, umbilical catheterization procedure, PVT location, risk factors, treatments, and long-term outcomes.

**Results:**

Median age of the patients at diagnosis was 10 days (range 3–90 days), and 69.6% of patients were girls. Of the 23 patients, 87% had left PVT and, 91.3% had at least one thrombosis risk factor, which was sepsis in 73.9% of patients, and presence of umbilical venous catheter in 87%. Totally, 59.1% of PVTs were completely resolved in a median follow-up of 7 months (1 month to 12 months), and 78.3% of these patients had no anticoagulant therapy (ACT). Partial thrombus resolution was achieved in 9 patients (40.9%). Five patients (%21) received ACT. Overall, 34.8% of patients had long-term complications. neonatal PVT is most commonly reported in the left portal vein and there is no evidence for the impact of ACT on reducing the short- or long-term complications. Well designed and larger studies are necessary to clarify this issue, which can facilitate developing appropriate management algorithms.

**Conclusion:**

Neonatal PVT is most commonly reported in the left portal vein and there is no evidence for the impact of ACT on reducing the short- or long-term complications. Well designed and larger studies are necessary to clarify this issue, which can facilitate developing appropriate management algorithms.

## Highlights

The overall incidence rate of PVT was 0,79/1000 neonatal intensive care admissions, possibly an underestimation of the incidence of true PVT. UVC insertion and sepsis were the leading potential risk factors for PVT.

Among 23 infants, both of neonates showed partial or complete PVT resolution with 9(%39) and 14(%61) respectively, within 18,4 days (4 days to 4 months). In both premature and term neonates the PR and CR rates were similiar.(40% and 60% versus 37% and %63).

In ACT recipients group (ACT group) and ACT non- recipients group(No ACT group), PR or CR mean duration were 38,6 days (8 days to 4 months) and 12,6 days (4 days to 3 months) respectively. This information may show us that the benefit of ACT therapy in the resolution of PVT is doubtful, and that wait-and-watch management should be prioritized in the profit-loss relationship.

11 articles were included in the systematic review of literature. Total 183 neonates 102 were ACT untreated and 81 were ACT treated. Overall PR and CR are displayed on Table 4.

Long-term complication rate was 8/23(%34) with mean 7 months(1 to 12 months) follow-up duration.

## Introduction

The incidence of neonatal portal vein thrombosis (PVT) has been reported as 1 in 10,000 live births and in 36 of 1000 neonatal intensive care (NICU) admissions [1]. Neonatal PVT is generally asymptomatic and in case of clinical suspicion, diagnosis is made by abdominal doppler ultrasound (USG) [2–4] Major risk factors for PVT in neonates include umbilical vein catheterization (UVC), sepsis, and thrombophilia. Neonatal UVC advances to the inferior vena cava through the umbilical vein, portal sinus, and ductus venosus, respectively [5, 6]. However, as a result of anatomical variants or incorrect applications, different levels can be advanced at the portal sinus level, usually the portal vein. [7]. This situation creates susceptibility to PVT through vascular endothelial damage and occlusion. [8]. It is important to diagnose and follow-up neonatal PVT due to long-term complications leading to fatal conditions such as portal hypertension (PHT) and gastrointestinal bleeding [8, 10]. Patients with unresolved thrombosis may develop Portal HT in the second decade of life. Today, neonatal risk factors for the development of PHT are not well known. There is no definite information on whether anticoagulant therapy (ACT) helps to resolve thrombosis or reduce the development of PHT [11–13]. This study aimed to determine risk factors, short and long-term consequences and evaluating treatment options in newborns with PVT [14].

## Materials and methods

In this study; 23 newborns diagnosed with PVT who were hospitalized at the Neonatal Intensive Care Unit of Health Science Univercity Basaksehir Cam and Sakura Hospital, Istanbul, between 2020 and 2022 were retrospectively analyzed. Demographic data of patients, including median age at diagnosis, gender and median follow up, umbilical catheterization data, underlying disease, catheter placement sites, PVT location, prenatal risk factors, birth types; coagulation tests including PT, APTT, INR and D-dimer, liver function tests including alanine transaminase (ALT) and aspartate transaminase (AST), C-reactive protein, genetic thrombophilia test results, thrombosis detection time by Doppler ultrasound, treatment methods and durations, thrombosis follow-up and thrombosis examinations were retrospectively reviewed from the patients’ files. Subjects with a birth weight of less than 10th percentile for gestational age were defined as small for gestational age (SGA), and the birth of a baby less than 37 weeks of gestational age was defined as preterm birth. Neonatal period was defined as 28 days of life at term and up to 44 weeks of gestational age in preterm babies. Patients diagnosed with PVT at neonatal period were included in the study. Neonates who did not meet these criteria were excluded from the study. Portal hypertension was defined as the presence of either clinical or radiological evidence of splenomegaly, presence of portosystemic collateral veins, and abnormal portal vein flow. Portal vein thrombosis diagnosed by doppler abdominal ultrasound, and the confirmation of PVT diagnosis and treatment decision was made by a pediatric hematologist. Patients were also followed up for long-term complications. Informed consent was obtained from the parents of all participating subjects.

### Review of the literature

Articles published between 2011 and 2021 that describe PVT and ACT use /or not in neonates were identified in a systematic review. Using the keywords “neonatal”, “infant-newborn”, “PVT”, “anticoagulant”, “enoxaparin”, “heparin”, “treatment and outcome”; electronic literature search was performed from MEDLINE, EMBASE, PUBMED and OVID medical websites, provided that the diagnosis was limited to < 28 days of age. Studies were included if they reported ACT use or not for noenatal PVT. All studies were checked for references to additional studies, and each article identified from the database was compared, to verify matching and exclude duplication. The correspondance author first reviewed the abstracts to confirm eligibility, and selected articles were processed for full-text analysis. Any discrepancies identified were discussed with a second author and a consensus decision was reached.

### Statistical analysis

Statistical analyses were performed using IBM SPSS V23. Demographic characteristics were described using mean ± standard deviation and median (minimum – maximum) for quantitative variables, and categorical data was described with number and percentage. Normality assumption of the data was evaluated with the Shapiro-Wilk Test. Paired Samples T-test and/or Wilcoxon test was used for comparison two dependent groups according to normality of data distribution. Cohra’s Q Test was used for comparison within groups. All statistical tests were two-sided, and the level of statistical significance was set at P < 0.01.

## Results

**Fig. 1a-1b:**
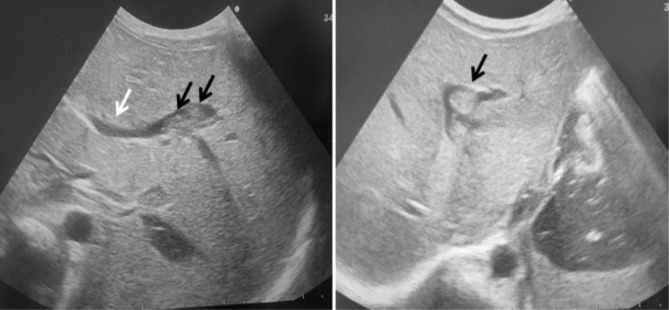
**1a** Partial thrombus allowing flow in the left portal vein lumen after UVc (black arrow), **1b**- right portal vein is patent(white arrow)

**Fig. 2:**
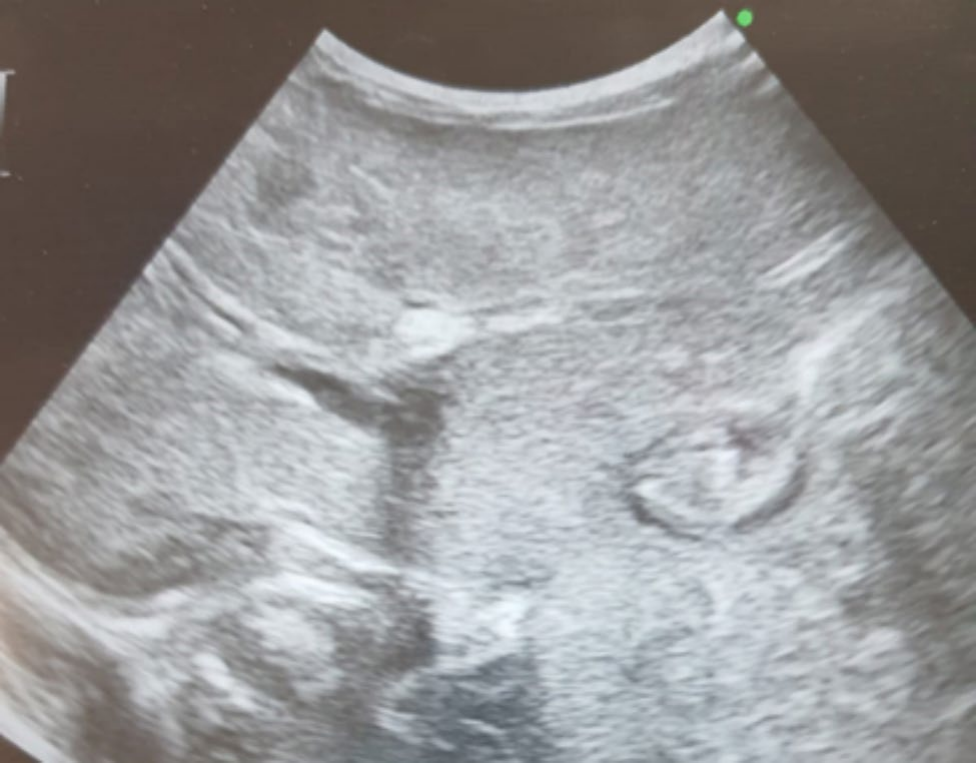
An echogenic thrombus (black arrow) with a posterior acoustic shadow in the left portal vein lumen after UVC. In the proximal part of the right portal vein, the lumen is open (white arrow) with a thrombus in the lumen distally harmonious increase in echogenicity (dashed arrow), liver parenchyma is observed heterogeneously

**Fig. 3:**
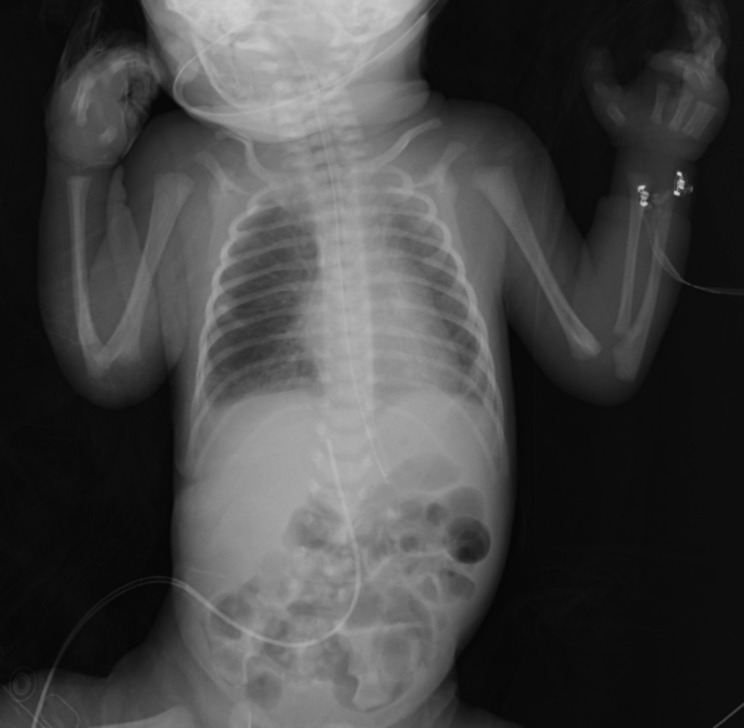
A 33 gestestational age new born with inserted nasogastric sandoge and UVC

There were 16 female (69.6%) and 7 male (30.4%) patients. The median age at diagnosis was 10 days (range 10–90 days), and median follow up time was 7 months (range 1–12 months). Fifteen patients were preterm, and 8 patients were term baby. Median gestational age of 23 patients was 36 weeks (range 24–41 weeks). Demographic data of the patients and characteristic features of thrombosis are summarized in Table 1. The site of thrombosis was portal vein in 87% of patients, and 91.3% had at least one thrombosis risk factor, which was sepsis in 73.9% of patients, and presence of umbilical venous catheter in 87%. Anticoagulant treatment was given in 5 (21.7%) patients, and 78.3% of patients had no ACT. Median duration of ACT treatment mean was 14 days (8–41 days). Genetic tests were not performed in 90.9% of patients. Totally, 59.1% of PVTs were completely resolved in a median follow-up of 7 months (1 month to 12 months), and 78.3% of these patients had no anticoagulant therapy. Overall in 1 year follow up; 8 patients (34.8% ) had long-term complications (Table 2). Partial thrombus resolution was achieved in 9 patients (40.9%). Complet and partial thrombus resolution rate for term baby was 60%, and 40%, respectively, whereas 63%, and 37% for preterm baby, without any significant difference (Table 3) (p < 0,01). Median leukocyte count of the patients was 10.73 × 10^9^/L, followed by a median hemoglobin of 13.29 g/dl, a median platelet of 296.91 × 10^9^/l. Laboratuatry features of the patients are presented in Table 4. As the current study aimed to review literature of neonatal PVT, results of 11 review among 51 articles that meet inclusion criteria for the systematic review are showed in Table 5. The radiological findings of UVC localization and ultrasonographic findings of PVT in three separate patient are illustrated in Figs. 1, 2 and 3.

**Table 1:** Patients caharacteristics and thrombosis features

Age at diagnosis, median, days	10 (range 10–90)	
Follow up time, median, months	7 (range 1–12)	
Gestesional week of birth, median, weeks	36 (range 24–41 )	
Birth weight, median, gram	2800 (range 1156–4360 )	
	N	%
Birth Path		
C/S (cesarian insio)	16	69.5
Spontaneous vaginal	7	30.5
Gender		
Female	16	69.6
Male	7	30.4
PVT Risk factors		
UVC	21	91.3
Sepsis	17	73.9
Spontaneous	1	4.3
Polycthemia	1	4.3
Site of thromosis		
PV	20	87,0
UV/IVC	1	4,3
UV/PV	1	4,3
UV	1	4,3
Detalied site of thrombosis		
Left PV	8	34,8
Left PV median	2	8,7
Left PV distal	1	4,3
Left PV confluex	1	4,3
Left PV bifurcation	1	4,3
UV extension to PV confluex	1	4,3
UV extension to left PV	1	4,3
Left PV superior	1	4,3
Left PV superior medial	1	4,3
Left PV	1	4,3
UV extension to IVC	1	4,3
Left PV extension to umblical confluex	1	4,3
UV extension to left PV superior	1	4,3
PV bifurcation	1	4,3
Other	1	9,3

**Table 2:** Thrombosis and treatment outcomes

	Frequency (n)	Percentage (%)
Follow-up time		
< 1 month	1	4,3
3 months	6	26,1
6 months	9	39,1
9 months	2	8,7
12 months	5	21,7
Resolution		
Complet	13	59,1
Partial	9	40,9
Antitrombolitic Treament		
Negative	18	78,3
Positive	5	21,7
Plasma Factor Levels		
No info	18	81,8
Normal	1	4,5
Abnormal (F8:%46,F9%25,F11%42)	3	13,6
Genetic (thrombophilia panel)		
No info	20	90,9
Negative	2	9,1
Long term complication ( 12 months follow-up)		
Negative	15	65,2
Positive	8	34,8

**Table 3:** Thrombosis resolution and long-term complication of infants by gestational age

Characteristic	Preterm (< 37GH) N = 15	Term (> 37GH) N = 8	All N = 23	P < 0,01
**Resolution rate, n, %**				
Partial	6 (40%)	3(37%)	9(39%)	p > 0,01
Complet	9(60%)	5(63%)	14(61%)	
**Complications**				
Extension	1(0.06%)	1(0.12%)		p > 0,01
Portal HT	0 (0%)	1(0.12%)		
Other*	1(0.06%)	4(50%)	8(34%)	

*Other: Hepatic atrophy, gastrointestinal system bleeding, hepatic venous thrombosis, splenomegaly

**Table 4:** Laboratory results of patients

	Median (min - max)
WBC (10^9/l)	11.5 (2.9–19)
Haemoglobin (g/dl)	14 (7.9–20)
Platelet (10^9/l)	273 (72–557)
ALT(u/l)	14.5 (6–144)
PT (sec)	10.1 (8.9–19)
aPTT (sec)	39.2 (21.8–69)
CRP (mgr/dl)	1.7 (0–118)
D-Dimer (µg/ml)	1.6 (0.97–5.82)

**Table 5:** Systematic review of the literature for outcomes of neonatal portal vein thrombosis in the management and follow-up management (2010–2022)

Study-year-(ref) N of cases-UVC related	Treatment CR-PR	Other
**Gharehbagi MM et al.-2011-(8 )** 5 − 4 (%80) UVC related	0 3 CR/2 Death (3–6 weeks)	Mean GA:30 weeks %80 UVC
**Morag L et al.-2011-(9)** 133 − 95(%74) UVC related	59(%44 ACT) Not stated	Only 70 patients followed median of 55 months (range, 24–96). 14 (20%) normal; 2 (3%) PHT; 18 (26%) LLA; 5 (7%) splenomegaly.
**Korber F et al.-2011-(10)** 14 − 1 (%7) UVC related	2(%16 ACT) 1 CR with ACT, 1 recanalized spontaneous	14 (6 term; 8 preterm [24–35 weeks]); 13 occlusive, 1 nonocclusive. 11untreated had increased liver arteries.
**Hery G et al.-2013-(11)** 3- UVC unrelated	1(%33 surgical) Not stated	3 cases GA: a34 weeks ,†37 weeks, ‡39 weeks (all occlusive). None UVC related. iAt 9 years: portal cavernoma, PHTN, splenomegaly † At 9 years: portal cavernoma, PHTN, esophageal varices At 2.5 years: normal portal flow.
**Pergantau H et al.-2014-(12)** 7- UVC unrelated	4(%57ACT) 5CR(4 treated,1 Spontaneous)	At median of 6 years :5 (71%) CR (4 treated; 1 untreated); 1 (14%, untreated) PHTN; 1 (14%, untreated) esophageal varices
**Rios Mendez RE et al.-(13)** 1- UVC related	1(%100 ACT) CR	Follow-up for 2.5 months and reported normal
**Himami F et al.-2014-(14)** 1- UVC unrelated	0 Not stated	At 18 months: portal cavernoma persisted
**Cakir U et al.-2015-(15)** 1-UVC related	1(UFH/LMWH CR for 8 weeks)	1 term infant. Nonocclusive, post TAPVR surgery. At 6 months: No portal venous thrombosis or PHTN
**Rambaund G et al.-2015-(16)** 1- UVC related	0 CR	1 case. 34 weeks; nonocclusive; UVC related; associated Tetralogy of Fallot
**Gluffre M et al.-2016-(17**) 1- UVC related	1(LMWH for CR 1 month)	32 weeks GA appears occlusive but not stated. At 1 year: PR
**Salih CC et al. -2020-(18)** 13- (5 complet and 8 partial occlusion)	13 13 CR (7–120 days (LMWH for 31 ± 13.8 days)	Mean GA: 29 ± 2 weeks No additional data for follow ups.

Total N = 183; total treated (n = 81); total untreated (n = 102); thrombus resolved (n = 27); thrombus unstated status(n = 137)

Abbreviations: ACT( anticoagulant therapy); GA(gestational age); LMWH(low-molecular-weight heparin); PHT( portal hypertension); PVT(portal venous thrombosis); TAPVR(total anomalous pulmonary venous return); UFH(unfractionated heparin); UAC(umbilical arterial catheter); UVC( umbilical venous catheter)

## Discussion

There is an increased risk of PVT in newborns, especially in prematures. The most important reason for this is UVC for the use of venous vascular access, especially in premature newborns [15, 16] . Moreover; procoagulants, anticoagulants, and fibrinolytic pathway proteins can be found at rates up to 50% lower than in adults, depending on the gestational week of birth [17–19]. The risk of PVT increases further due to endothelial damage and physiological inbalance after UVC insertion. Left PV is the main thrombosis site as in our study [20, 21]. Park and et al. reported the UVC-related PVC rate as 75% in a total of 57 cases between 1948 and 2012. Similarly, in our study this ratio was 87%.

The other main risk factors for PVT include prematurity (PM), low birth weight, sepsis, perinatal asfixia, thrombophilia, congenital heart disease, prolonged UVK, blood product transfusion and thrombophilia mutations such as F V Leiden, methylenetetrahydrofolate reducatese (MTHFR) in most studies. In our study, sepsis was present in 73.9% of patients with a median CRP of 17.81 mg/L (range 0 to 118 mg/dl) at diagnosis. Median gestational week of delivery, and median birth weight was 34.95 weeks (range 24 to 41 weeks), and 2585.3 g (range 1156 to 4360 g), respectively, which were consistent with previous data in literature. Unfortunately; factor level analysis and genetic data was not available in 91.3%, and in 90.9% of our patients, respectively. This was one of our study’s limitation, however, there is no data of genetic tests and coagulation profile in most studies in the literatüre either. There were only 2 neonates with negative results, who were screened for thrombophilia mutations (F V Leiden, MTHFR, prothrombin G20210A, antithrombin III, protein C, protein S and plasminogen activator inhibitor). Four patients were screened for coagulation factor levels and 3 of 4 were revealed abnormal results as follow: Factor VIII level: 46%, FIX level: 25%, FXI level:42%, but the relationship between mildly low coagulation factor levels and PVT in newborns was not determined. Advanced researchs with large number of patients are need to define relation between the thrombophilia /genetic factors and neonatal PVT.

D-dimer blood testing, clinical evaluation, and ultrasound imaging are generally used in the evaluation of suspected deep vein thrombosis (DVT) in the lower extremities [18]. Deep vein thrombosis can be ruled out by evaluation that includes a low clinical presumption, negative D-dimer test (< 0.5 µg/ml) and negative ultrasound imaging of the proximal veins [22]. In the current study, the median value of D-dimer for 9 neonates with PVT was 1,6 µg/ml (0,97 to 5,82 µg/ml ) at diagnosis, which was higher than normal D-dimer ranges, suggesting that D-dimer levels may be used as a diagnostic tool and in the evaluation of response to treatment in neonatal PVT. However, since there is no data on D-dimer values ​​in newborns without portal vein thrombosis in our institution, the importance of D-dimer elevation in the diagnosis and treatment follow-up in this line group could not be determined. Also, septicemia as a confounding factor, may lead to elevated D-dimer levels, and this should be kept in mind while evaluating the high D-dimer levels in neonatal PVT.

Surfactant use is a well known factor leading to side effects such as coagulation disorders, decreased platelet count, and disseminated intravascular coagulation [23]. In our study; 9 patients received endotracheal surfactant due to respiratory distress syndrome. Further studies are needed to identify the significance of these prenatal risk factor on neonatal PVT.

There is no standard management algorithm for neonatal PVT treatment yet. The wait-and-watch strategy is generally used for neonatal PVT management [24]. In accordance to this approach, wait-and-watch strategy was used in this study. Low molecular weight heparin (LMWH) is the first choice in the presence of occlusion or enlargement of affected vein detected by doppler USG and clinical symptoms. Studies on recombinant tissue plasminogen activator or urokinase therapy in complicated thrombosis have been reported. But there is few data about the benefit of thrombolytic agents use. In our clinical practice, we used LMWH, at a dose of 2 × 100 unit/kg /per day, in selected 5 patients. The ACT treatment plan of all newborns with PVT was made by council of pediatric hematology and neonatology divisions of the institute, according to the portal vein involvement branch, lumen obstruction rate, extension and liver function tests. Median duration of ACT was 14 days (8–41 days), and CR rate was achieved in 3 of 5 patients (%60) and PR rate was achieved in 2 of 5 (%40) in the ACT group, respectively. In No ACT group, CR and PR ratio was %55 (10/18 patients), and 45% (8/18 patients), respectively. There was no significant difference for CR/PR ratios between both untreated and ACT groups. Our results were similiar with Moreg et al. study [24].

PVT is usually asymptomatic in the neonatal period, hepatic atrophy may occur after PVT without impairment in liver function tests. In children; PVT is the most common cause of extrahepatic PHT and GIS bleeding. The main cause of PVT in the neonatal period is umbilical vein catheterization [25] In our study, long-term complication ratio was 34% (8/23 patients) in a median 7 months (1–12 months) follow-up. Of the 8 patients with long-term complication, 1 (4.3%) developed PHT, 1 developed extension of thrombosis to other portal vien branches, 3 patients (13%) had hepatic parenchyma complications, and 3 remaining patients (13%) had hepatic vein thrombosis. In ACT group non of the patient had GIS bleeding symptoms due to treatment. In Morag et al. study; in a median follow up of 55 months (range 24–96 months), PVT-related long-term complications were PHT (2 patients, 3%), left liver lobe atrophy (18 patients, 26%), and splenomegaly (5 patients, 7%) [24]. Similarly, PHT ratio was lower in the current study compared to the latter study. (%3 versus 4.3%). Longer follow up is necessary to determine the risks for long-term complications in neonatal PVT.

Outcomes of neonatal PVT in the short and long term are seem to be related to the degree of occlusion of affected vein and extent of thrombus within the hepatic venous circulation and the liver parenchyma involvement [25, 26]. In addition, PR/CR status and median duration of thrombosis resolution are the other significant factors in terms of better outcome achievement. In the present study, long-term complication rate was significantly higher in term babies, which was 75% in 8 term babies and 20% in 15 preterm babies, respectively. There was no significant direct effect of preterm or term delivery on the development of neonatal PVT in our study. The impact of C/S delivery on neonatal PVT is unclear. The high rate of C/S delivery in our study was due to the predominance of premature cases.

Premature babies need long-term venous access due to the long hospitalization duration. In this regard, UVC is widely used in these patients for administration of drugs, parenteral nutrition, fluids, and blood products. Therefore, premature babies seem to have increased risk for neonatal PVT [27, 28].

Median follow up of ACT for neonatal PVT is reported between 3 months and 55 months in the literature (Table 4). Median duration of follow up for our study cases was 7 months (1 to 12 months). Significant long-term consequences of neonatal PVT that causes morbidity and mortality are liver lobe atrophy, portal hypertension, esophageal varices, portal collaterals and liver cavernoma. These complications can be diagnosed more easily depending on the length of follow-up [28, 29]. Mihir D.bhatt et al. studied 44 preterm and 30 term infants with PVT and showed that 51.7% of obstructive PVT resolved completely spontaneously [29]. In line with this; in our study, 78.3% of PVT completely resolved spontaneously; this raises the question of which infant group truly deserves ACT or thrombolysis to avoid serious future complications, including those with thrombi that have progressed or enlarged from the main portal vein to other branches.

There are some limitations in our study. First, follow-up durations of patients was short due to variable causes. All newborns with PVT in the institute follow-up are examine at least weekly and at most monthly according to the patient’s clinic after discharge. Liver function tests and portal USG are routinely performed in newborns with PVT who come to this follow-up period. It is very difficult to continue regular follow-up of these patients due to the fact that they generally do not come to outpatient follow-up after discharge because of epidemic situations such as the coronavirus pandemic, economic and other social reasons. The second limitation is that genetic thrombophilia screening for our study could not be performed on every patient due to financial and institutional reasons. A genetic research panel should be established and new studies should be conducted to better evaluate the etiology and risk factors that predispose to PVT in newborns.

The multidisiplinary approach will be highlighten the underlying factors moreover management strategies in neonatal PVT.

## Conclusion

The Left Portal Vein is the main site for thrombosis risk. Proper UVC application can reduce the risk of PVT. As a result of the evaluation of the literature; provided information that short-term and long-term prognosis can be improved with early-term appropriate ACT treatment. Also watch-and-wait management can be considered in indivudals especially portal vein thrombosus as ACT treatment seems uneffective. However, neonatal diagnosis and treatment of PVT need to be standardized with more prospective clinical studies.

## Data Availability

Not applicable.

## References

[1] Morag I, Epelman M, Daneman A (2006). Portal vein thrombosis in the neonate: risk factors, course, and outcome. J Pediatr.

[2] Williams S, Chan AK (2011). Neonatal portal vein thrombosis: diagnosis and management. Semin Fetal Neonatal Med.

[3] Junker P, Egeblad M, Nielsen O, Kamper J (1976). Umbilical vein catheterization and portal hypertension. Acta Paediatr Scand.

[4] Sethi SK, Dewan P, Faridi MM, Aggarwal A, Upreti L (2007). Liver abscess, portal vein thrombosis and cavernoma formation following umbilical vein catherisation in two neonates. Trop Gastroenterol.

[5] Shah I, Bhatnagar S (2009). Liver abscess in a newborn leading to portal vein thrombosis. Indian J Pediatr.

[6] El-Karaksy H, El-Koofy N, El-Hawary M (2004). Prevalence of factor V Leiden mutation and other hereditary thrombophilic factors in egyptian children with portal vein thrombosis: results of a single-center case-control study. Ann Hematol.

[6] Ferri PM, Rodrigues Ferreira A, Fagundes ED (2012). Evaluation of the presence of hereditary and acquired thrombophilias in brazilian children and adolescents with diagnoses of portal vein thrombosis. J Pediatr Gastroenterol Nutr.

[7] Morag I, Shah PS, Epelman M (2011). Childhood outcomes of neonates diagnosed with portal vein thrombosis. J Paediatr Child Health.

[8] Gharehbaghi MM, Nemati M, Hosseinpour SS, Taei R, Ghargharechi R (2011). Umbilical vascular catheter associated portal vein thrombosis detected by ultrasound. Indian J Pediatr.

[10] Körber F, Demant AW, Schulze Uphoff U, Kabbasch C, Lackner KJ (2011). Occlusion of the left portal vein in newborns. Ultraschall Med.

[11] Héry G, Quarello E, Gorincour G, Franchi S, Gauthier F, de Lagausie P. Extrahepatic vitelline vein aneurysm: prenatal diagnosis and follow up. J Pediatr Surg. 2013 Aug;48:e1–e4. 10.1016/j.jpedsurg.2013.06.007 23932633

[12] Pergantou H, Avgeri M, Komitopoulou A (2014). Venous thromboembolism at uncommon sites in neonates and children. J Pediatr Hematol Oncol.

[13] Ríos-Méndez RE, Giménez P (2014). Intracardiac persistence of pericatheter fibrin sheath in a newborn: case report. Arch Argent Pediatr.

[14] Hmami F, Oulmaati A, Mahmoud M, Boubou M, Tizniti S, Bouharrou A (2014). Neonatal group a streptococcal meningitis and portal vein thrombosis: a casual association. Arch Pediatr.

[15] Çakır U, Kahvecioglu D, Alan S (2015). Portal vein thrombosis of a new- ˘ born with corrected total anomalous pulmonary venous return. Turk J Haematol.

[16] Rambaud J, Grévent D, Bergounioux J (2015). Portal vein thrombosis and stroke in a patient with tetralogy of Fallot. J Pediatr Gastroenterol Nutr.

[17] Giuffrè M, Verso CL, Serra G, Moceri G, Cimador M, Corsello G, Study Group of Neonatal Infectious Diseases Affiliated to the Italian Society of Neonatology (2016). Portal vein thrombosis in a preterm newborn with mutation of the MTHFR and PAI-1 genes and sepsis by candida parapsilosis. Am J Perinatol.

[18] Salih Çağrı (2020). Hilal Özkan,Bayram Ali Dorum, Nilgün Köksal, Pınar Kudretoğlu, Birol Baytan, Melike Sezgin and Adalet Meral Güneş. The danger awaiting premature babies: portal vein thrombosis. Turk Pediatri Ars.

[19] Geersing GJ, Zuithoff NPA, Kearon C (2014). Exclusion of deep vein thrombosis using the Wells rule in clinically important subgroups: individual patient data meta-analysis. BMJ.

[20] Wells PS, Anderson DR, Bormanis J (1997). Value of assessment of pretest probability of deep-vein thrombosis in clinical management. Lancet.

[21] Wells PS, Owen C, Doucette S, Fergusson D, Tran H. Does this patient have deep vein thrombosis?JAMA 2006;295:199–207. doi:10.1001/jama.295.2.199. 10.1001/jama.295.2.199 16403932

[22] Wells PS, Anderson DR, Rodger M (2003). Evaluation of D-dimer in the diagnosis of suspected deep-vein thrombosis. N Engl J Med.

[23] A Proposed Role of surfactant in platelet function and treatment of Pulmonary Hemorrhage in Preterm and Term Infants. Acta Haematol. 2018;140(4):215–20. Epub 2018 Oct 19. 10.1159/000493082 30343298

[24] Morag I, Epelman M, Daneman A (2006). Portal vein thrombosis in the neonate: risk factors, course, and outcome. J Pediatr.

[25] Thompson EN, Sherlock S (1964). The aetiology of portal vein thrombosis withparticular reference to the role of infection and exchange transfusion. Q J Med.

[26] Mihir D, Bhatt V, Patel,Michelle L, Butt, Anthony KC, Chan B. Paes. Outcomes following neonatal portal vein thrombosis: a descriptive, single-center study and review of anticoagulant theraphy.Pediatric blood and Cancer.18 Ekim 2018. 10.1002/pbc.27572 30520242

[27] Pacifico L, Panero A, Colarizi P, Matrunola M, Simonetti AF, Chiesa C (2004). Neonatal Candida albicans septic thrombosis of the portal vein followed by cavernous transformation of the vessel. J Clin Microbiol.

[28] Demirel N, Aydin M, Zenciroglu A, Bas AY, Yarali N, Okumus N (2009). Neonatal thrombo-embolism: risk factors, clinical features and outcome. Ann Trop Paediatr.

[29] Mihir D, Bhatt V, Patel,Michelle L, Butt, Anthony KC, Chan B. Paes. Outcomes following neonatal portal vein thrombosis: a descriptive, single-center study and review of anticoagulant theraphy.Pediatric blood and Cancer.18 oct 2018. 10.1002/pbc.27572 30520242

